# Multiscale Cosine Convolution Neural Network for Robust and Interpretable Epileptic EEG Detection

**DOI:** 10.3390/bios16040203

**Published:** 2026-04-02

**Authors:** Jiale Chen, Weidong Zhou, Guoyang Liu

**Affiliations:** 1 School of Integrated Circuits, Shandong University, Jinan 250101, China; 202300400062@mail.sdu.edu.cn (J.C.); wdzhou@sdu.edu.cn (W.Z.); 2 Shenzhen Research Institute of Shandong University, Shenzhen 518000, China

**Keywords:** seizure detection, multiscale convolutional neural network, cosine convolution, Heterogeneous Two-Stream Network, EEG signal processing

## Abstract

The accurate detection of epileptic seizures using an electroencephalogram (EEG) is essential for clinical diagnosis and reducing the burden on clinicians but remains challenging due to low detection performance and model interpretability. In this study, we propose a Multiscale Cosine Convolutional Heterogeneous Two-Stream Cosine Convolution Network (MCC-HTSCC) to overcome these limitations. First, the raw EEG signals are input into the Multiscale Cosine Convolution (MCC) module, where multiscale temporal features are extracted by cosine convolutional layers with varying kernel lengths. Subsequently, the extracted temporal features are further processed through spatial convolutional layers to obtain comprehensive spatiotemporal representations. These spatiotemporal features are fused and subsequently fed into the Heterogeneous Two-Stream Cosine Convolution (HTSCC) module, comprising both deep and shallow subnetworks to perform hierarchical feature extraction and classification. Extensive evaluations were conducted on the publicly available CHB-MIT dataset and a clinically collected SH-SDU dataset, achieving accuracies of 98.52% and 94.56%, sensitivities of 97.98% and 88.09%, and specificities of 98.50% and 95.89%, respectively. Furthermore, the cosine convolution operators reduce the learnable parameters of our model by approximately 18.12% compared to the model with traditional convolution operators, making it more suitable for embedded deployment. By employing the Gradient-Weighted Class Activation Mapping (Grad-CAM) technique, we further provide interpretability and transparency in model decision making, highlighting the substantial potential of MCC-HTSCC for effective patient-specific epilepsy monitoring and diagnostics.

## 1. Introduction

Epilepsy is a chronic neurological disorder characterized by recurrent seizures arising from abnormal, hypersynchronous neuronal activity in the brain [1]. Electroencephalography (EEG), which measures cortical electrical activity recorded from the scalp, remains a cornerstone for the diagnosis and management of epilepsy [2]. The global burden of epilepsy is substantial. The World Health Organization (WHO) reports that around 50 million people live with epilepsy worldwide, while population-level estimates can vary across regions and studies due to differences in case definitions, ascertainment strategies, and data coverage [3,4]. Epidemiological estimates of epilepsy prevalence vary across settings and are strongly influenced by survey design and diagnostic criteria. For example, population-based evidence synthesized in a systematic review of studies conducted in China indicates that epilepsy remains an important public health challenge, while burden analyses further suggest substantial regional heterogeneity [5]. Because seizures may occur unpredictably, patients can face elevated risks of injury or other adverse outcomes, particularly when events occur without timely assistance or in hazardous settings. In clinical practice, routine EEG assessment often relies on an expert visual review, in which neurologists identify seizure activity and interictal epileptiform discharges from recordings. Although EEG provides direct evidence of abnormal electrical patterns, reviewing prolonged or continuous EEG streams is labor-intensive and time-consuming, which can limit efficiency and increase the likelihood of missed events, especially under heavy clinical workloads [6]. These challenges motivate the development of accurate and efficient automated EEG-based seizure detection systems to support clinical decision making and improve monitoring efficiency.

The current seizure detection methods can be roughly categorized into threshold-based and classification-based techniques. Threshold-based methods offer strong interpretability and are straightforward to implement but often suffer from poor generalization. This approach was first introduced by Gotman [7], who developed the initial automatic epileptic EEG detection system by decomposing EEG signals into half-waves and extracting morphological features of rhythmic brain activity to identify seizures. Despite being more efficient than expert visual inspection, threshold-based methods exhibit significant variability between patients and sensitivity to threshold adjustments, often resulting in high false positive rates [8]. To address these limitations, researchers have introduced several hand-crafted features, such as diffusion distance [9], the spike rate [10], common spatial patterns (CSP) [11,12], power spectral density (PSD) [13,14], and fractal-based methods [15,16]. These features have improved discrimination and epileptic EEG detection accuracy. Nevertheless, they have notable drawbacks, including high sensitivity to preprocessing, susceptibility to individual variability and noise interference, and dependence on manual feature selection, limiting robustness in complex detection scenarios. Additionally, feature applicability can vary significantly between patients and datasets, constraining their generalization performance. With advancements in machine learning techniques, classification-based methods have emerged as more advantageous alternatives. Mursalin et al. [17] proposed a random forest-based classifier, significantly enhancing performance compared to traditional threshold methods. Other researchers have introduced approaches utilizing neural fuzzy networks [18] and support vector machines (SVMs) [19], achieving improved accuracy. However, despite their superior precision, machine learning methods still face challenges related to limited generalization capability.

Deep learning, as an advanced branch of machine learning, has demonstrated superior performance attributed to its hierarchical architecture and powerful automatic feature extraction capabilities [20]. By utilizing deep neural networks in an end-to-end learning fashion, deep learning models can automatically extract high-level representations from complex, nonlinear data, substantially enhancing predictive accuracy and robustness. Consequently, deep learning has emerged as a promising solution to overcome the limitations inherent in conventional machine learning methods, particularly in medical diagnostics such as EEG-based seizure detection [21]. With significant advances in both theoretical frameworks and computational resources over the past decade, deep learning methods have achieved remarkable success across diverse application fields. Among various deep learning architectures, convolutional neural networks (CNNs) stand out due to their exceptional abilities in automatic feature extraction and pattern recognition, currently representing state-of-the-art techniques in numerous computer vision tasks. Recently, CNN-based methodologies have gained significant attention in automatic epileptic EEG detection due to their outstanding capacity for EEG data analysis and seizure prediction. Previous studies have demonstrated that CNN architectures offer considerable potential for enhancing the accuracy, robustness, and generalization performance of epileptic EEG detection systems. Chen et al. [22] proposed a CNN-based automated seizure detection approach, wherein EEG signals were first decomposed using Discrete Wavelet Transform (DWT) to derive diverse entropy-related features, including approximate entropy (ApEn), fuzzy entropy (FuzzyEn), sample entropy (SampEn), and standard deviation (STD). Subsequently, a random forest algorithm was employed to select representative features, which were then classified using a CNN model, achieving high precision in seizure detection. Similarly, Nie et al. [23] introduced a hybrid classification framework integrating fully convolutional network (FCN) and long short-term memory (LSTM) architectures. Their method converted raw EEG signals into frequency domain representations using Fast Fourier Transform (FFT), capturing essential temporal dynamics and spectral features for accurate classification. Recently, Anita et al. [24] introduced a novel epileptic EEG detection framework combining multiscale Atrous-based deep convolutional neural networks (MSA-DCNN) with LSTM networks for EEG-based seizure detection. Among these, the LSTM network is highlighted for its exceptional ability to capture the long-range temporal dependencies inherent to EEG time series data, effectively extracting dynamic temporal patterns associated with epileptic seizures, which substantially improved the model’s seizure detection accuracy. Recent investigations have further indicated that incorporating time frequency transformations into CNN-based pipelines significantly enhances the detection efficacy. For instance, Shen et al. [25] employed Short Time Fourier Transform (STFT) to convert raw EEG signals into time frequency images, which were subsequently classified using the GoogleNet architecture, leading to improved seizure detection performance. Moreover, Amiri et al. [26] presented an innovative automatic seizure detection method that initially extracted spatial features via Sparse Common Spatial Pattern (SCSP) and subsequently employed Adaptive Short Time Fourier Transform-based Synchrosqueezing Transform (ASTFT SST) to obtain high-resolution time frequency representations. Additionally, Zhong et al. [27] proposed a novel approach leveraging Stockwell Transform (S Transform) for accurate time frequency representation extraction from raw EEG segments. In their framework, the resulting time frequency matrices were grouped into distinct EEG rhythmic sub-blocks and compressed into informative feature vectors. These vectors were then input into a transformer-based network for efficient feature selection and classification. Collectively, these sophisticated methodologies provide more discriminative representations of EEG time frequency features, significantly bolstering the accuracy and robustness of seizure detection systems. Recently, Liu et al. [28] introduced a novel epileptic EEG detection approach leveraging the Stockwell transform (S-transform) to simultaneously extract phase and power spectra information from EEG signals. Their proposed method strategically enables convolutional neural networks (CNNs) to effectively capture the pronounced inter-channel phase synchronization characteristic of epileptic seizures, while concurrently enhancing the network’s sensitivity to both low-frequency and high-frequency spectral features within EEG data sourced from the CHB-MIT and Bonn databases. The study conclusively demonstrated that the inclusion of EEG phase information substantially enriches seizure-related discriminative features, underscoring the critical role that phase synchronization plays in epileptic event detection. Furthermore, the integration of phase-derived inputs significantly guided the CNN classifiers’ attention toward pertinent low-frequency and high-frequency spectral dynamics, thus markedly improving seizure classification accuracy and robustness. Despite the promising results achieved by CNNs and related deep learning frameworks, traditional CNN architectures typically demand substantial memory resources and computational power. Moreover, deeper CNN models often incur increased optimization difficulty and higher latency footprints, which further restrict deployment in resource-constrained and real-time settings. Such limitations critically restrict their deployment in resource-constrained scenarios that demand real-time and energy-efficient analysis, such as EEG-based epilepsy monitoring systems. Consequently, the practical implementation of CNNs in epileptic EEG detection imposes stringent requirements on computational efficiency, model robustness, and generalization capabilities.

To meet such efficiency constraints, two research lines are particularly relevant. One line focuses on lightweight CNN architectures (depthwise separable/group convolutions and bottleneck designs) to reduce computation through architectural factorization [29,30,31]. The other line explores structured or parameterized convolution kernels, where filters are constrained by analytic forms (sinusoidal/Gabor/Sinc or wavelet-inspired parameterizations) to improve parameter efficiency and inductive bias [32,33,34,35]. While both directions can reduce model complexity, their generic designs are not tailored to the time frequency characteristics of epileptic EEG, and they typically do not provide a task-specific mechanism to exploit structured kernels for multiscale temporal modeling under limited seizure data.

Motivated by the structured kernel CNN paradigm, previous work introduced the Cosine Convolutional Neural Network (CosCNN) to improve parameter efficiency by constraining convolutional filters to cosine basis functions [36]. Distinct from traditional CNN architectures, which typically require learning a substantial number of convolutional kernel parameters, the CosCNN leverages specially designed convolutional filters constructed from cosine basis functions. Each cosine convolution kernel depends solely on two learnable parameters, compressing the number of trainable parameters and substantially alleviating memory overhead and computational redundancy, thereby achieving superior computational efficiency. CosCNN has exhibited significant performance advantages in various signal processing fields [36,37,38]. Therefore, this study also incorporates the cosine convolutional operator and extends it with the design of lightweight, computationally efficient neural network models suitable for resource-constrained biomedical applications. However, existing cosine-kernel seizure detectors such as CosCNN primarily adopt single-scale feature extraction, which limits their ability to explicitly differentiate heterogeneous epileptiform characteristics that manifest across multiple temporal scales in a long-term scalp EEG [36,37,38]. Compared with previous single-scale cosine-kernel designs, our key difference is to introduce a multiscale temporal feature extraction scheme so that complementary epileptiform patterns at different receptive field scales can be captured within the same lightweight framework.

In this study, we extend previous single-scale cosine-kernel designs to a multiscale formulation and propose a novel architecture that integrates a Multiscale Cosine Convolution (MCC) module and a Heterogeneous Two-Stream Cosine Convolution (HTSCC) module for EEG-based seizure detection. By explicitly modeling temporal patterns at multiple receptive field scales with cosine parameterized kernels, the proposed network enhances discriminability under limited seizure data while retaining high computational efficiency. The proposed architecture effectively captures complex spatial–temporal dependencies inherent in EEG data, optimizes computational efficiency, and significantly improves model robustness and generalization under the patient-specific setting. The primary contributions of this work are summarized as follows:

1. We propose an MCC module that explicitly extends previous single-scale cosine-kernel EEG detectors to a multiscale formulation, enabling the network to capture epileptic EEG patterns at multiple temporal receptive field scales with a compact cosine parameterized kernel set. This design improves discriminability and patient-specific generalization under limited seizure data while maintaining low storage and computational cost.
2. We introduce a novel HTSCC module designed to comprehensively extract multiscale EEG features, substantially enhancing the robustness and computational efficiency of the model. This dual-stream heterogeneous design allows the network to achieve strong detection performance on two independent patient-specific EEG cohorts.
3. Extensive experimental evaluations performed on both the publicly available CHB-MIT epileptic EEG dataset and our clinically collected SH-SDU database demonstrate the proposed model’s outstanding performance, validating its efficacy and superiority in epileptic seizure detection tasks. Additionally, we utilize Gradient-weighted Class Activation Mapping (Grad-CAM) [39] to visualize and interpret the decision-making process of the network, significantly improving the interpretability and transparency of the proposed model.

The remainder of this paper is organized as follows. Section 2 provides a detailed introduction to the proposed Multiscale Cosine Convolution Heterogeneous Two-Stream Cosine Convolution (MCC-HTSCC) architecture, including specific implementations of the MCC and HTSCC modules. Section 3 describes the experimental datasets, data preprocessing methods, and experimental settings. Section 4 presents the experimental results, analyzes the model’s performance on the epileptic seizure detection task, and compares it with existing approaches. Finally, Section 5 concludes the paper by summarizing the main contributions and outlining directions for future research.

## 2. Materials and Methods

**Figure 1:**
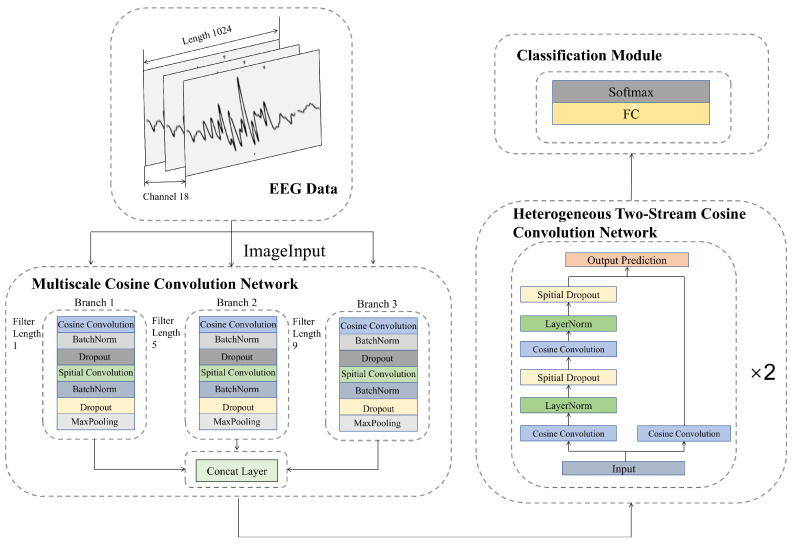
Overall architecture of the proposed MCC-HTSCC network. It comprises three parallel convolutional branches for initial feature extraction, two HTSCC modules, and a classification module. Each convolutional branch combines cosine convolutions and standard convolutions, followed by normalization, dropout, and maxpooling layers. The HTSCC module includes a shallow single-layer convolutional stream and a deeper stream with dual convolutional layers, each followed by LayerNorm and SpatialDropout. Finally, features are classified using fully connected and softmax layers.

In this section, we present an MCC-HTSCC network, as illustrated in Figure 1. The proposed network consists of three primary components, an MCC module, an HTSCC module, and a classification module.

### 2.1. Preprocessing

In this study, we employed two datasets, namely the publicly available CHB-MIT dataset [40] and the clinically collected SH-SDU dataset. The CHB-MIT database contains annotations for a total of 184 seizure events. In our experiments, 40 seizure events were selected as the training set, and the remaining 144 seizure events were utilized for testing, a data division strategy that has been widely adopted in multiple previous studies on the CHB-MIT benchmark [36,41,42]. For most patients, we employed their first seizure event as training data. However, for Patients 6, 12, 13, and 16, whose seizures were shorter but occurred more frequently, we selected 4–8 seizure events for training. To ensure multi-channel consistency across subjects, we used 18 common bipolar EEG channels shared by all CHB-MIT recordings (aligned with the international 10–20 system) and segmented all channels synchronously, so that each sample is represented as a multichannel EEG segment $E\in R^{C\times T}$ with C=18. Each continuous EEG recording was divided into fixed length segments of 4 s (T=1024 points at 256 Hz). Segment boundaries were determined on the time axis and applied identically to all channels to preserve temporal alignment. Segment labels were assigned according to expert-provided seizure annotations, segments overlapping annotated seizure intervals were labeled as seizure, whereas the remaining segments were labeled as non-seizure.

To mitigate data scarcity and class imbalance in a reproducible manner, we constructed a patient-specific training set by applying fixed sliding-window segmentation with five-fold overlap to the selected training seizure events. We also collected interictal training data whose total duration is approximately five times the seizure duration before segmentation. Specifically, we set the oversampling rate to $n_{\mathrm{over}}=5$. For ictal training data, we used a 4 s window of length T=1024 samples and a step size of $\Delta =\lfloor T/n_{\mathrm{over}}\rfloor =204$ samples, which corresponds to about an 80% overlap.

For each patient, we first concatenated the selected training seizure events across all channels to form a continuous ictal signal and then segmented it using a 4 s sliding window (1024 samples at 256 Hz) with a step of 204 samples, which corresponded to 80% overlap. This five-fold overlapping upsampling was applied only to the training seizure events to increase the number of ictal training segments. To prevent leakage, no segments were generated from any test seizure event, and the seizure events reserved for testing remained strictly held out for final evaluation.

For interictal training data, we did not apply overlap augmentation. Instead, we extracted $2n_{\mathrm{over}}$ seizure-free raw EEG chunks from non-ictal periods (uniformly spaced over the available seizure-free indices), each with length $\approx L_{\mathrm{sz}}/2$, and concatenated them to form an interictal training stream with total duration $\approx 2n_{\mathrm{over}}\times \left(L_{\mathrm{sz}}/2\right)=n_{\mathrm{over}}L_{\mathrm{sz}}$, approximately five times the duration of the collected training seizure signal. This interictal stream was then segmented into non-overlapping 4 s windows (step *T*) to produce non-seizure training segments.

In contrast, the testing set was kept in its original chronological order without any overlap-based augmentation, so that the reported segment-based and event-based results reflect long-term continuous monitoring. In addition, we extracted an extra 20 min seizure-free EEG segment from each patient as a calibration dataset, which was used to patient specifically calibrate the post-processing parameters (the MAF smoothing window *N*, decision threshold Thr, and collar length *K*) for score smoothing, thresholding, and collar refinement in event-level detection. Importantly, this calibration set was not used for network training and did not update any model weights, and it was strictly separated from both the training and testing sets by selecting non-ictal periods that do not overlap with any seizure events or non-seizure segments used for training/testing and by excluding it from any sample generation or upsampling procedure. Moreover, no additional signal-level normalization (z-score scaling) was performed across patients or globally in preprocessing. Instead, raw segment amplitudes after filtering were retained, while Batch Normalization layers inside the network were used to stabilize training. Ultimately, our training dataset comprised 3.04 h of EEG recordings, while the testing dataset included the remaining 976.89 h.

During EEG preprocessing, we applied DWT filtering with Daubechies-4 (Db4) wavelets [43] to mitigate noise and artifacts. The EEG signals were decomposed into five frequency sub-bands, d1 (64–128 Hz), d2 (32–64 Hz), d3 (16–32 Hz), d4 (8–16 Hz), d5 (4–8 Hz), and a low-frequency approximation component a5 (0–4 Hz). Considering the clinical relevance to seizure events, frequency bands from 4 to 32 Hz (sub-bands d3, d4, d5) were selected for further analysis. The same preprocessing procedure was applied to the SH-SDU dataset to ensure consistency between the two datasets.

### 2.2. Multiscale Cosine Convolution

**Figure 2:**
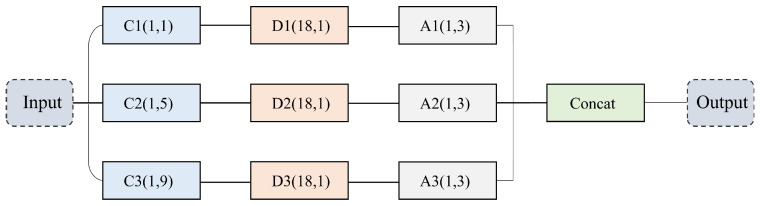
Detailed structure of the proposed MCC module. C1, C2, and C3 represent cosine convolution layers. D1, D2, and D3 denote spatial convolution layers, and A1, A2, and A3 indicate maxpooling layers. Each convolutional layer is followed by Batch Normalization and Dropout layers.

EEG signals encompass diverse time frequency patterns, and features at different scales can significantly influence the accuracy of epileptic EEG detection. As illustrated in Figure 2, we adopt a multiscale cosine convolution module composed of several parallel branches. Each branch employs cosine convolution kernels of different lengths, namely 9, 5, and 1 to capture multiscale EEG characteristics. This module utilizes three parallel convolutional branches equipped with cosine convolution kernels of varying lengths to extract multiscale temporal EEG features. Specifically, the branch with a kernel length of 9 is designed to capture low-frequency components of EEG signals, thereby modeling global trends that are particularly relevant for identifying broad epileptic activity. Meanwhile, the branch with a kernel length of 5 focuses on mid-level temporal features, effectively detecting local short-term dynamics and transient epileptic waveforms. By capturing these localized fluctuations, this branch enhances the ability to recognize short-duration seizure patterns. Lastly, a branch with a kernel length of 1 serves to compress features and facilitate channel-level information interaction, thereby strengthening the expressiveness of the overall representation. Outputs from these branches are concatenated to enhance feature representational power. Unlike standard convolutions, cosine convolutions rely only on two learnable parameters, amplitude (*A*) and frequency (ω), substantially reducing computational complexity while preserving sufficient modeling capacity for extracting relevant EEG patterns. A cosine convolution kernel of length *k* at position *m* is mathematically defined as:

(1) $$K_{m}\left(A,\omega ,k\right)=A\cos\;\left(\omega \left(m-\frac{k-1}{2}\right)\right),\;m\in \left\{0,1,\ldots ,k-1\right\}.$$

where k∈N denotes the kernel length, *m* is the discrete kernel index, A∈R is the amplitude, and ω∈R is the angular frequency. The centering term $\left(m-\frac{k-1}{2}\right)$ enforces a center-symmetric kernel.

It can be observed that the cosine convolution kernel exhibits a center-symmetric structure, rendering its convolution with the input signal mathematically equivalent to a cross-correlation. During model initialization, both the amplitude *A* and frequency ω are sampled from a Gaussian distribution with mean 0 and variance 1. This random initialization scheme ensures that the network starts from a wide range of potential kernel shapes and frequencies, thereby promoting more effective exploration of the EEG feature space during training. Importantly, this parameterization also explains the reduction in model size, unlike a conventional convolution kernel of length *k* that requires learning *k* independent weights, each cosine-kernel is fully determined by only two learnable parameters (*A* and ω), which generate all *k* kernel coefficients through the cosine function. Therefore, the number of learnable parameters per kernel is reduced from *k* to 2, yielding a reduction in k−2 parameters, a parameter reduction ratio of $\frac{k-2}{k}\times 100\%$.

### 2.3. Heterogeneous Two-Stream Cosine Convolution

**Figure 3:**
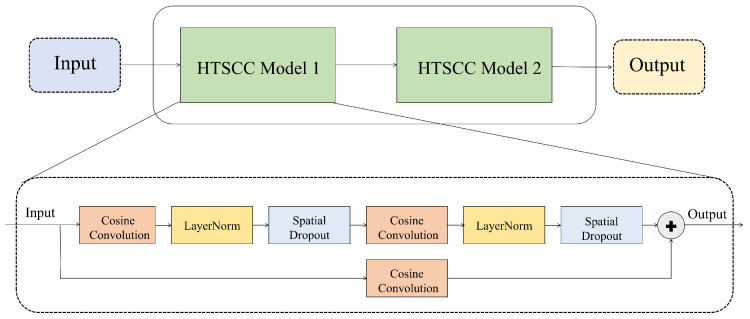
Architecture of the proposed HTSCC module.

To further enhance feature extraction from fused multiscale features, we propose the HTSCC module shown in Figure 3. This module comprises shallow and deep convolutional streams designed to capture features at different levels of abstraction within EEG signals. The shallow branch employs a single cosine convolution layer with a kernel length of 3. This branch omits normalization and regularization to directly capture lower-level temporal patterns, thereby preserving the raw characteristics of the EEG signal. The deep stream consists of two-layer cosine convolutions (kernel length = 3), each followed by layer normalization to stabilize training and spatial dropout to prevent overfitting, thus extracting higher-level abstract features, yielding a more robust representation of complex epileptic activity. Features from both streams are merged and forwarded to fully connected and softmax layers for seizure classification.

### 2.4. Postprocessing

**Figure 4:**
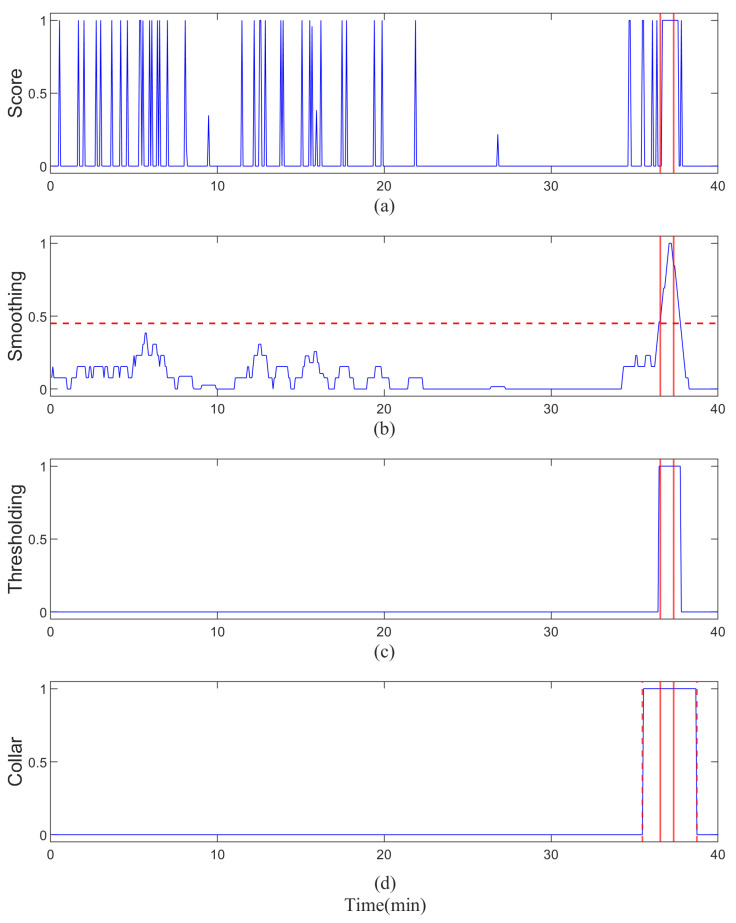
An example of post-processing 40 min model output scores. The blue line indicates the prediction scores. The data within two solid red lines correspond to the predicted seizure scores. (**a**) The raw scores obtained from the softmax layer. (**b**) The smoothed scores by utilizing MAF. The red dashed horizontal line denotes the threshold. (**c**) The binary decision data made with a fixed threshold. (**d**) The predicted label after adding the collar. The red dashed vertical line denotes the final decision.

In long-term epileptic EEG databases, raw EEG signals often contain substantial noise and artifacts acquired during data collection, thereby increasing the false alarm rate in automatic seizure detection. To enhance the stability and accuracy of the final model output, this work implements a post-processing pipeline based on the model’s predicted scores, comprising score smoothing, thresholding, and the collar technique. Specifically, the smoothing stage employs a moving average filter (MAF) to suppress isolated false positives. The MAF is defined as follows:

(2) $$y_{t}=\frac{1}{2N+1}\sum_{n=-N}^{N}x_{t+n},\;t\in \left\{N,N+1,\ldots ,T-N\right\}.$$

where $x_{t}$ denotes the predicted seizure score at time index *t*, $y_{t}$ is the smoothed score, N∈N is the half-window size (thus, the window length is 2N+1), and *T* is the length of the score sequence. Where $x_{t+n}$ represents the softmax score at time t+n, and $y_{t}$ denotes the smoothed score at time *t*. The resulting smoothed output was then thresholded using a predefined parameter, *Thr*, to generate binary decision labels, with values greater than *Thr* classified as seizure (label 1) and values below the threshold labeled as non-seizure (label 0). While this smoothing strategy is effective at suppressing isolated false positives, it introduces a degree of temporal blurring at seizure boundaries, potentially delaying the precise localization of onset and offset. The full post-processing pipeline applied to a 40 min output sequence is illustrated in Figure 4.

To mitigate this temporal imprecision, we further introduced a collar refinement strategy. This involves extending both the onset and offset of detected seizure segments by *K* units in time, effectively broadening the detected window to account for boundary uncertainty and better capture the true extent of seizure activity. The post-processing parameters, namely *N*, Thr, and *K*, serve as patient-specific operating settings to control the sensitivity-specificity trade-off and were tuned individually for each patient using a calibration segment, while the test set was reserved solely for final performance reporting with these parameters fixed. After that, the post-processing operations [36], including smoothing, thresholding, and collar, are employed to remove isolated false detections and improve the sensitivity and specificity of the detection results.

## 3. Results

### 3.1. Datasets

#### 3.1.1. CHB-MIT EEG Database

Model training and evaluation were conducted on the CHB-MIT scalp EEG database [40], which contains continuous EEG recordings from 24 pediatric epilepsy patients. The original recordings contain 18–23 bipolar channels aligned with the international 10–20 placement system. To ensure consistency of model input across patients while making full use of the available recordings, 18 common bipolar channels shared across the selected data were used in this work [12,36,44]. These channels were FP1-F7, F7-T7, T7-P7, P7-O1, FP1-F3, F3-C3, C3-P3, P3-O1, FP2-F4, F4-C4, C4-P4, P4-O2, FP2-F8, F8-T8, T8-P8, P8-O2, FZ-CZ, and CZ-PZ.

Regarding data acquisition, the CHB-MIT recordings were collected in a clinical long-term monitoring setting at the Children’s Hospital Boston. Each patient underwent continuous scalp EEG monitoring over multiple days, typically after the withdrawal of anti-seizure medication, with the clinical goal of capturing and characterizing spontaneous seizures and assessing candidacy for surgical intervention [40]. The signals were recorded at 256 Hz with 16-bit resolution.

To provide a concise database description, we additionally summarized each subject’s demographic information (e.g., patient index, sex, and age), seizure phenotype (SP/CP/GTC), and clinically annotated seizure onset zone (temporal/frontal/occipital), as well as the total EEG recording duration and the mean seizure duration in Table 1. This table clarifies the composition and heterogeneity of the 24-patient dataset and facilitates reproducibility and cross-study comparison when interpreting the patient-specific training/testing results. Patient selection and eligibility were determined by the database itself, rather than by additional subject-level screening in our study. Instead, we used all 24 pediatric subjects provided in the CHB-MIT database for whom the required scalp EEG channels and expert seizure annotations were available.

**Figure 5:**
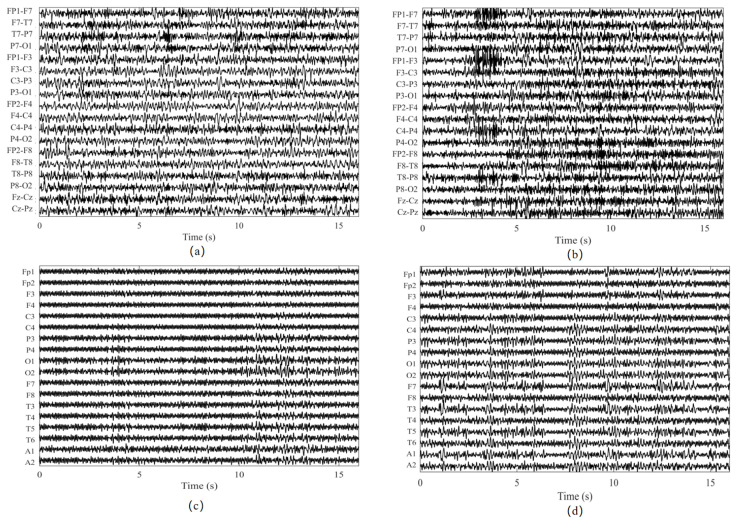
Representative multi-channel EEG segments from two datasets. Panels (**a**,**b**) are from the CHB-MIT dataset, where panel (**a**) shows a non-seizure (normal) segment, and panel (**b**) shows an ictal (seizure) segment. Panels (**c**,**d**) are from the SH-SDU dataset, where panel (**c**) shows a non-seizure (normal) segment, and panel (**d**) shows an ictal (seizure) segment.

For an intuitive illustration of the CHB-MIT signals used in this study, Figure 5a,b shows two representative 18-channel scalp EEG segments from the CHB-MIT database, where panel (a) corresponds to a non-seizure (normal) segment, and panel (b) corresponds to an ictal (seizure) segment. This visualization provides a direct qualitative contrast between background activity and seizure-related patterns in CHB-MIT recordings, complementing the database statistics reported in Table 1.

#### 3.1.2. SH-SDU EEG Database

We further validated our model on a more clinically challenging EEG dataset collected at the Second Hospital of Shandong University (SH-SDU), affiliated with Shandong University. The SH-SDU recordings were acquired during routine clinical care for epilepsy diagnosis and pretreatment evaluation. Patients were admitted for long-term scalp EEG monitoring (clinical EEG/long-term monitoring ward), and EEG was continuously recorded throughout hospitalization to capture spontaneous seizure events under medical supervision. The synchronized clinical records and EEG traces were reviewed after acquisition. Seizure events were identified and time-stamped by experienced clinicians to establish the ground-truth annotations used in this study. This dataset consists of long-term scalp EEG recordings from 10 adult patients with epilepsy and exhibits a higher seizure frequency than the CHB-MIT dataset. Table 2 summarizes the SH-SDU database and recording statistics at the patient level, including sex, age, the total annotated recording duration, mean seizure duration, and the number of EEG channels used. All subjects were recorded using the international 10–20 system with 18 unipolar electrodes, including ear electrodes A1 and A2, with the reference electrode (G) placed at the midpoint between Fz and Cz. The selected derivations include Fp1, Fp2, F3, F4, C3, C4, P3, P4, O1, O2, F7, F8, T3, T4, T5, T6, A1, and A2. The recordings were not manually preselected or trimmed before analysis, and potential artifacts (e.g., EMG/EOG contamination) were mitigated through signal preprocessing. A total of 148.23 h of EEG recordings were annotated in the SH-SDU dataset, containing 143 seizure events confirmed by at least two neurologists.

Compared with the CHB-MIT pediatric epileptic EEG database, patients in the SH-SDU database are adults and exhibit a relatively higher seizure frequency. During model testing, for most patients, the initial one or two seizure events were chosen as training sets, and subsequent seizure episodes served as testing sets. To avoid train-test leakage, the split was performed at the seizure event-level (training seizure events and testing seizure events were strictly disjoint), and continuous non-ictal periods were segmented accordingly under the same rules. For patients with unusually short or long seizures, the number of seizure events assigned to training was increased or decreased within a predefined range, while maintaining the same event-level separation principle, and the per-patient split statistics are reported in Table 3 and Table 4.

For an intuitive illustration of the SH-SDU signals used in this study, Figure 5c,d shows two representative 18-channel scalp EEG segments from the SH-SDU database, where panel (c) corresponds to a non-seizure (normal) segment and panel (d) corresponds to an ictal (seizure) segment. This visualization provides a direct qualitative contrast between background activity and seizure-related patterns in SH-SDU recordings, complementing the database statistics reported in Table 2.

### 3.2. Experimental Setup

All experiments were performed in the Matlab R2024b environment, running on a 13th Gen Intel^®^ Core^TM^ i7-13700H CPU, an NVIDIA GeForce RTX 4060 Laptop GPU, and 16 GB of RAM.

Neural network training in this study was conducted within the MATLAB Deep Learning Toolbox environment. We employed the trainnet function with the cross-entropy loss function. The Adam optimizer was used, with an initial learning rate of $2\times 10^{-4}$ and a final learning rate of $2\times 10^{-5}$. A piecewise learning rate decay strategy was implemented, reducing the learning rate by a factor of approximately 0.89125 every 20 epochs. The model was trained for a total of 500 epochs with a batch size of 128, performing a validation step after each mini-batch. Early stopping was not enabled. The training data were randomly shuffled at the start of each epoch, and visualization features were disabled during training. All training was executed on a GPU platform.

This study evaluates the seizure detection performance of MCC-HTSCC on the CHB-MIT database using three assessment methods, segment-based, event-based, and the area under the Receiver Operating Characteristic curve (AUROC, hereinafter referred to as AUC). In patient-specific tasks, the MCC-HTSCC model is independently trained and tested for each patient, that is, each patient’s data are partitioned into a dedicated training set and testing set, with no cross-patient mixing. This setup assesses the personalized detection capabilities of the model. In segment-based evaluation, the classification performance is measured by sensitivity, specificity, and accuracy. Sensitivity reflects the model’s ability to detect seizure segments, specificity indicates its capacity to correctly reject non-seizure segments, and accuracy denotes the overall classification correctness. In event-based evaluation, the event-based sensitivity and false detection rate (FDR) are used to gauge the practical utility of seizure event detection; the former measures whether seizure occurrences are captured by the model, whereas the latter quantifies the frequency of false alarms. To further assess model performance under a unified post-processing setting, we additionally introduce the AUROC metric and apply a uniform smoothing window and collar time window in post-processing. Specifically, when calculating AUROC, the smoothing window length and collar length are fixed at 24 s for all patients. In addition, because AUC is a threshold-independent metric, it reflects classifier performance across all possible decision thresholds, rather than relying on a single fixed Thr. Finally, based on these evaluation settings, the individualized detection results for all 24 patients are summarized, and the average performance metrics are presented to provide a comprehensive assessment of the potential of MCC-HTSCC for patient-specific seizure detection.

### 3.3. Results on CHB-MIT Database

As is illustrated in Table 5, under segment-based evaluation, the proposed MCC-HTSCC achieves an average sensitivity of 97.98%, an accuracy of 98.52%, and a specificity of 98.53%. Notably, perfect (100%) sensitivity was observed in 17 patients, and 14 patients exhibited specificity greater than 99%. In the event-based evaluation scenario shown in Table 6, our model successfully detected 142 out of 144 seizure events, yielding a low FDR of 0.95 events/h and an impressive average event-based sensitivity of 98.61%. All patients, except Patient 13 and Patient 18, achieved event-based sensitivity of 100%, and remarkably, the FDR for 11 patients was below 0.1 events/h. For robustness, the overall results are reported as mean ± standard deviation (mean ± SD), where the SD is computed across the 24 patients in the dataset.

In addition, we observed that Patient 16 (FDR > 10/h) and Patient 6 (FDR > 3/h) present relatively higher false detection rates compared with the majority of subjects. A plausible explanation is that these patients exhibit very short-duration yet highly frequent seizures, with multiple events occurring within brief time windows. Such patterns make seizure onsets/offsets less distinct, increasing the likelihood that transient non-seizure fluctuations are erroneously elevated to seizure alarms and thereby inflating the FDR. Finally, the high intra-patient variability of short seizures, reflected in substantial waveform and spectral differences across events, further challenges consistent discrimination, collectively leading to more isolated false detections and consequently elevated FDRs for Patients 16 and 6. In particular, Patient 16 is more severely affected by limited ictal training data. As shown in Table 1, their mean seizure duration is only 8.40 s (the shortest among all subjects) with relatively frequent events, resulting in fewer informative ictal segments and insufficient effective training iterations. This makes it harder for the model to internalize stable patient-specific ictal signatures and reliably distinguish them from brief non-seizure transients, which in turn increases the FDR.

Based on Table 7, the three seizure-type groups show different trade-offs between detection and false alarms. The SP/CP group provides the strongest false alarm control, with the lowest FDR (0.11/h) and the highest specificity and accuracy (both 99.58%), but its event-based sensitivity is lower (95.00%), indicating a higher risk of missed events. The SP/CP/GTC group achieves higher event-based sensitivity (99.04%) with strong segment sensitivity (97.79%), but this comes with reduced specificity (98.07%) and a higher FDR (1.16/h), implying a larger alarm burden. The CP/GTC group reaches perfect segment-based and event-based sensitivity (both 100.00%), suggesting highly detectable seizures; yet, it also exhibits the highest FDR (1.47/h) and lower specificity (98.41%), reflecting the greatest propensity for false alarms. Overall, when handling SP/CP seizures, the model tends to be conservative and prioritize false alarm suppression, whereas, for SP/CP/GTC and especially CP/GTC seizures, it tends to favor capturing seizure events more aggressively, which can increase false detections. Table 7, therefore, provides a phenotype level view that complements the patient-level segment-based and event-based summaries in Table 5 and Table 6.

### 3.4. Results on SH-SDU Database

To further validate our model’s robustness and clinical feasibility in a more challenging clinical scenario, we conducted additional evaluations on the SH-SDU dataset using identical metrics to those applied on the CHB-MIT dataset. As summarized in Table 3, our model achieved an average segment-based sensitivity of 88.09% and specificity of 95.89% across all ten patients. Importantly, seven patients exhibited both sensitivity and specificity greater than 85%. Additionally, the overall average classification accuracy reached 94.56%, underscoring the feasibility and reliability of our model in processing real-world clinical epileptic EEG data within this dataset. For robustness, the overall results are reported as mean ± standard deviation (mean ± SD), where SD is computed across the 10 patients in the dataset.

An event-based evaluation on the SH-SDU dataset, as summarized in Table 4, further demonstrates our effectiveness, among 143 seizure events, the model accurately detected 131 events, achieving an average FDR of 0.62 events/h Notably, Patient 8, despite experiencing a particularly high seizure frequency (37 seizures), exhibited an event-based sensitivity of 100%, illustrating the robust performance of our model under high seizure load conditions. Even with inherent limitations, such as a relatively small training data size and significant EEG artifacts, the model achieved a perfect event-based sensitivity (100%) in six out of the ten patients. We emphasize that these results reflect patient-specific training and calibration within a limited-size clinical database. Thus, broader cross-patient or cross-dataset generalization warrants further validation.

## 4. Discussion

To thoroughly validate the effectiveness of the proposed MCC-HTSCC network, we design and conduct an ablation study to assess the contribution of each core module to the overall model performance. Specifically, the Multiscale Cosine Convolution module and the HTSCC module are removed separately to construct several variant models. These variants are evaluated under the same experimental settings, and their final performance is compared using the AUC values of Patient 12. Patient 12 was chosen because their training and test sets contain the largest number of seizure events in the CHB-MIT database, thereby providing a relatively more sufficient basis for ablation comparison. Through this process, the effectiveness of each proposed module is further verified.

### 4.1. Ablation Studies

#### 4.1.1. Effect of the Number of Branches

To systematically verify the effectiveness of each scale within our proposed multiscale cosine convolution framework, we conducted controlled experiments with Patient 12, who had the highest number of seizures in the CHB-MIT dataset, using network variants with incremental convolutional branches. Specifically, we started from a single branch configuration (Branch = 1), progressively adding parallel convolutional branches to either repeatedly extract features at the same temporal scale or to extract features across multiple temporal scales, thereby minimizing potential feature loss. To maintain strict control over variables, all convolutional branches within each variant shared identical convolutional kernel lengths, ensuring consistent temporal scales. Except for variations in the number of convolutional branches, all other architectural and training parameters were kept constant across experiments. This systematic design allowed us to precisely quantify the impact of introducing additional convolutional branches on the robustness and discriminative capability of extracted features.

As shown in Table 8, the proposed three-branch parallel cosine convolution network substantially outperforms the single-scale single-branch convolutional network. By repeatedly extracting features across similar frequency domains, the three-branch model notably improves classification accuracy, highlighting its superior capacity to represent EEG signals across multiple scales. Specifically, when each of the three parallel convolutional branches within the multiscale cosine convolution module utilized a kernel length of 1, classification accuracy initially improved as the number of branches increased, before subsequently declining. Concurrently, the number of model parameters increased linearly with the addition of convolutional branches. These observations suggest that introducing a moderate number of parallel convolutional branches effectively enhances the model’s ability to capture richer temporal and frequency domain characteristics from EEG data, thereby improving seizure detection performance. However, when the number of branches exceeds an optimal threshold, the resulting redundancy in extracted features increases model complexity and may lead to overfitting, ultimately degrading generalization capability, as reflected in the decline in classification accuracy.

#### 4.1.2. Effect of the Cosine-Kernel Lengths

**Figure 6:**
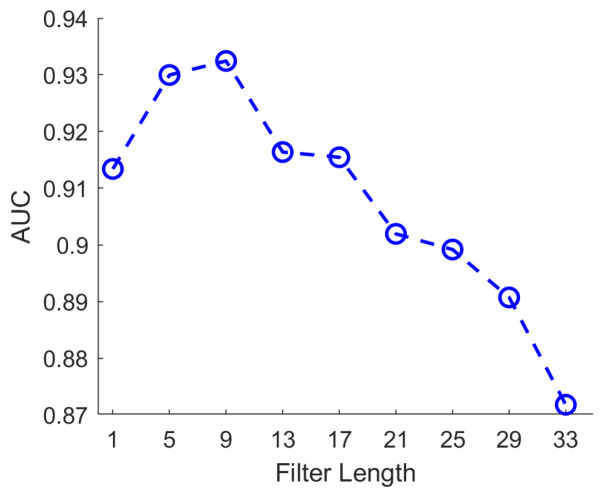
AUC performance under different filter lengths. The best AUC is achieved at filter length 9 (AUC = 0.932).

To further optimize convolutional kernel sizes and identify the most discriminative temporal scales for epileptic seizure detection, we conducted a systematic series of experiments designed to evaluate classification accuracy across various kernel lengths. In these experiments, kernel lengths across all three convolutional branches were incrementally increased, starting from 1 and progressing in steps of 4 (1, 5, 9, 13, 17), enabling a comprehensive assessment of each temporal scale’s contribution to model performance. All experimental conditions were carefully controlled to isolate the effect of kernel length, specifically, network architecture, training configurations, and hyperparameters were kept consistent throughout. To minimize the influence of random fluctuations inherent to the training process, each experiment was repeated multiple times, with the average AUC used as the primary evaluation metric. As shown in Figure 6, when all three branches share the same kernel length, classification accuracy initially increases with kernel length, peaks, and then gradually declines. Notably, kernel lengths of 1, 5, 9, 13, and 17 consistently yielded higher AUC scores, suggesting that these temporal resolutions are particularly effective for extracting EEG features indicative of epileptic events.

**Figure 7:**
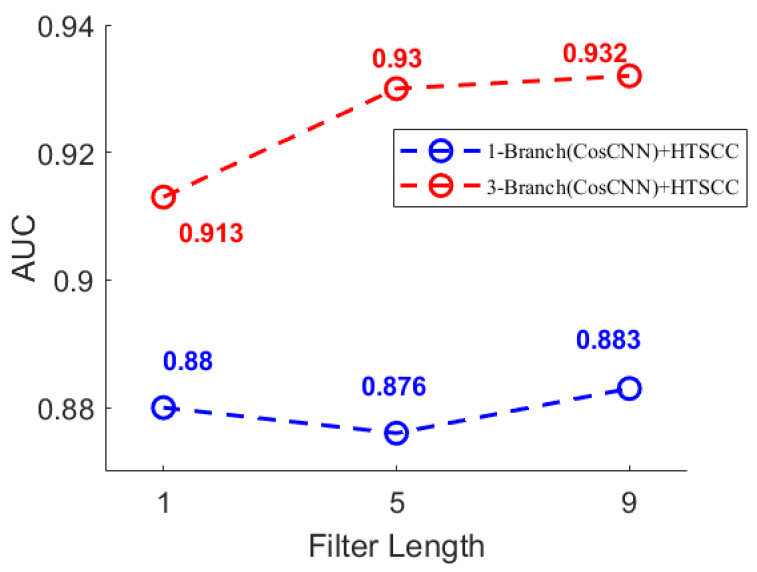
AUC comparison of 1-Branch(CosCNN) + HTSCC and 3-Branch(CosCNN) + HTSCC with varying filter lengths. The 3-Branch model consistently outperforms the 1-Branch model.

**Figure 8:**
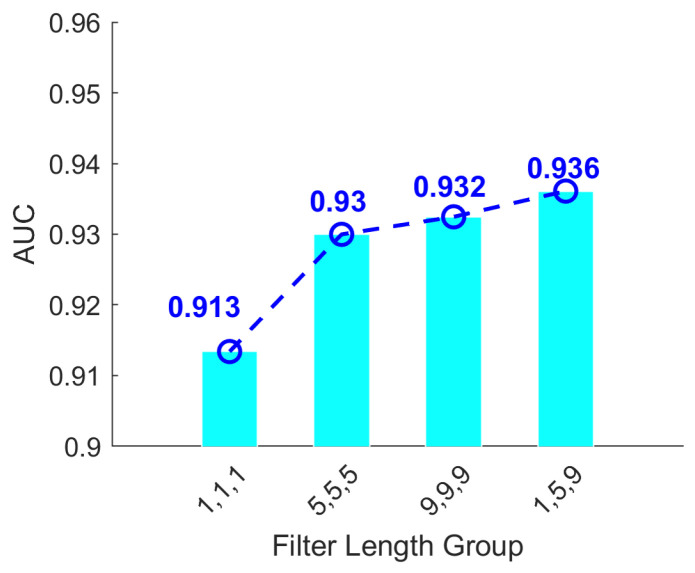
AUC scores under different filter group settings. The multiscale group (1, 5, 9) achieves the highest AUC = 0.936.

Building upon this finding, we further explored the impact of combining multiple temporal scales within a single model by assigning distinct initial kernel lengths to each convolutional branch. The kernel lengths in each branch were systematically varied in increments of 4, enabling a comprehensive evaluation of multiscale feature combinations. As clearly illustrated in Figure 7 and Figure 8, models that extract multiscale features consistently outperform those relying on repeated single-scale extractions. Specifically, Figure 7 compares single-branch and three-branch models, where the latter performs multiple extractions on the same scale, thereby mitigating information loss and improving classification accuracy. In contrast, Figure 8 examines models with identical parameter counts but different kernel length strategies. It reveals that employing varied kernel lengths across branches, thus extracting features at multiple scales, achieves higher average classification accuracy than using repeated kernels of the same size. These findings emphasize the effectiveness of multiscale feature integration in capturing diverse EEG characteristics critical for accurate epileptic seizure detection.

**Figure 9:**
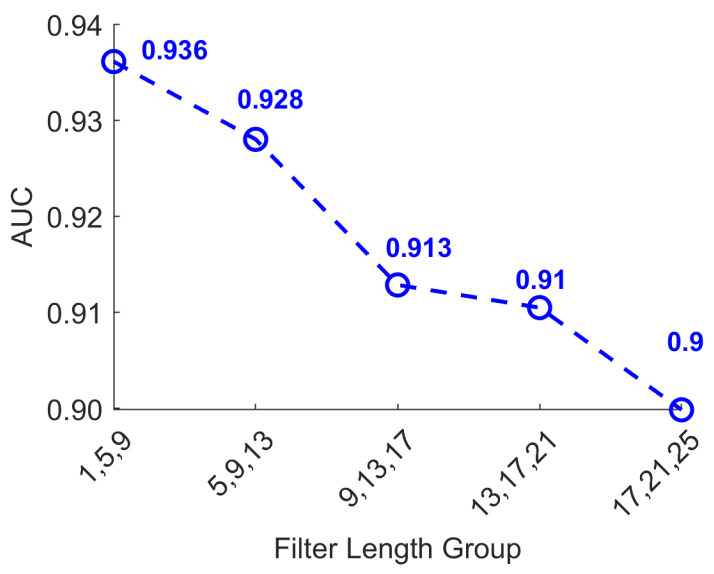
AUC scores under different filter lengths of multiscale filter groups. Performance decreases as the filter group shifts to larger lengths.

To further investigate the impact of multiscale feature combinations on seizure detection performance, this set of experiments builds upon the previously established finding that models extracting multiscale features consistently outperform those relying on repeated single-scale extraction. Specifically, distinct initial convolution kernel lengths were assigned to each of the three convolutional branches to explore the optimal kernel length configuration for each branch. As depicted in Figure 9, increasing kernel lengths across all three branches eventually led to diminishing returns in classification accuracy. In this experiment, each convolutional branch adopted a distinct kernel length, and the x axis denotes the specific combination of these kernel lengths. The objective was to identify the optimal multiscale frequency domain configuration for seizure detection. The results indicate that the model achieved its highest average classification accuracy when the three branches used kernel lengths of 1, 5, and 9. Beyond this configuration, accuracy gradually declined. This outcome strongly suggests that features extracted at these optimal temporal scales comprehensively capture critical EEG signal characteristics associated with epileptic seizures, thereby significantly enhancing overall classification performance.

#### 4.1.3. Effect of the HTSCC Module

**Figure 10:**
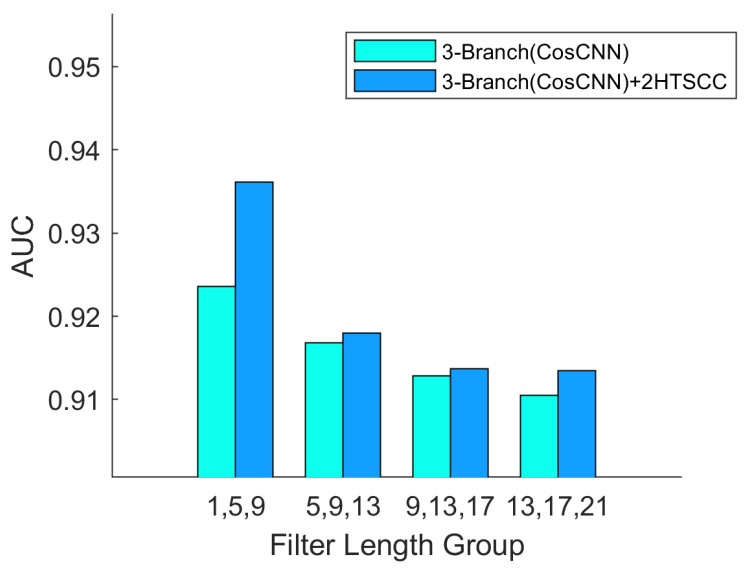
AUC performance of 3-Branch(CosCNN) vs. +2HTSCC across filter length groups. Adding HTSCC consistently improves AUC across the tested groups (the best setting reaches 0.936 with +2HTSCC).

To systematically verify the effectiveness of the proposed HTSCC module, we conducted controlled comparative experiments on Patient 12 from the CHB-MIT dataset, evaluating models with and without HTSCC integration. To ensure experimental rigor and objectivity, all conditions, except for the inclusion of the HTSCC module, were strictly kept constant, including network architecture, training configurations, and hyperparameters. Each experiment was repeated multiple times under both optimal and sub-optimal parameter settings, with the average AUC used as the primary evaluation metric. As illustrated in Figure 10, the x axis represents the convolution kernel lengths of the three parallel branches, while the two bar charts correspond to models with and without the HTSCC module. The comparative results clearly demonstrate that incorporating HTSCC significantly enhances seizure detection accuracy for Patient 12. The integration of HTSCC improves the model’s capacity to extract features at different levels of abstraction from EEG signals, leading to better classification performance. Under optimal parameter configurations, the addition of HTSCC improved accuracy by approximately 1.25%, outperforming all other experimental conditions. This empirical evidence strongly confirms the superior effectiveness of the proposed HTSCC module in capturing both spatial and temporal information from EEG signals, thereby enhancing the model’s generalization ability and overall detection performance in epileptic seizure recognition.

Building upon previous experimental evidence confirming the effectiveness of the HTSCC module, we conducted a more in-depth investigation to further assess its impact on epileptic seizure detection. Specifically, this set of experiments aimed to evaluate the performance contribution of each HTSCC module by incrementally varying the number of modules integrated into the network. Beginning with a baseline configuration containing a single HTSCC module, we progressively increased the module count while keeping the structural parameters of each instance identical. All other experimental conditions were strictly controlled, and each setup was repeated multiple times to ensure statistical reliability. The average AUC was employed as the primary evaluation metric to comprehensively assess classification performance and determine the optimal number of HTSCC modules. As summarized in Table 9, the results exhibit a clear trend, as the number of HTSCC modules increases, classification performance improves notably at first and then plateaus. In particular, performance saturates when the number of modules exceeds two, with AUC values consistently remaining above 92.5%. However, further increases in the module count lead to significant growth in model parameters, thereby elevating computational complexity and cost. Considering the trade-off between accuracy and efficiency, the final model adopts a two-module HTSCC configuration, which achieves an optimal balance between high classification accuracy and low computational overhead.

#### 4.1.4. Effect of the Cosine Convolution Module

In order to validate the effectiveness of the Cosine Convolution framework more explicitly, we replaced all standard convolutional layers, except for the spatial convolutional layer within the Multiscale Cosine Convolution module, with Cosine Convolution layers. The modified architecture was then trained using identical network hyperparameters and structural settings to ensure a fair comparison, and its performance was evaluated based on the average AUC on patient-specific EEG data (Patient 12 from the CHB-MIT dataset).

As shown in Table 10, incorporating Cosine Convolution layers led to an improvement in AUC by approximately 0.4% over the baseline model using only standard convolution. Meanwhile, the number of trainable parameters was significantly reduced by about 17.7 k. This corresponds to an 18.12% reduction compared to the original model with 97.7 k parameters. These results indicate that Cosine Convolution not only enhances detective performance but also reduces model complexity, offering substantial advantages for EEG-based epileptic seizure detection.

### 4.2. Visualization and Interpretability Analysis

#### 4.2.1. t-SNE Visualization

To intuitively evaluate the effectiveness of each proposed module, we performed an extensive visualization analysis based on t-distributed Stochastic Neighbor Embedding (t-SNE) [45]. Specifically, after fully training the MCC-HTSCC network, we selected Patient 12’s test dataset from the CHB-MIT database, sampling 200 normal segments and 200 seizure segments. We subsequently applied t-SNE dimensionality reduction separately to the raw input data, the outputs of the Multiscale Cosine Convolution module, and the final representations after the HTSCC module. The resulting visualizations provide clear and intuitive insights into the distinct contributions and efficacy of each network module.

**Figure 11:**
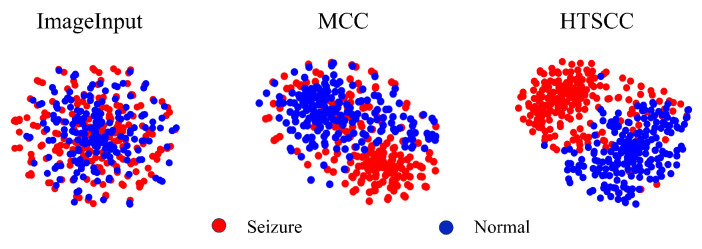
The feature vector clustering analysis using t-SNE. We randomly selected 200 seizure samples and 200 non-seizure samples from Patient 12. Each dot represents one segment, and the progressive separation indicates improved discriminability after MCC and HTSCC feature extraction.

Figure 11 presents the progressive evolution of the t-SNE distributions at different stages throughout network training. The visualizations clearly demonstrate a gradual separation of seizure and non-seizure clusters as training advances, highlighting the incremental enhancement of discriminative power at each processing stage. These results explicitly validate the significant roles of both the Multiscale Cosine Convolution and HTSCC modules in effectively extracting and distinguishing EEG features associated with epileptic seizures.

#### 4.2.2. Interpretability Analysis

While deep learning models can achieve exceptional detection accuracy, their inherent complexity often leads to a black-box nature, making it challenging to interpret and justify model decisions. This interpretability challenge arises predominantly from the intricate, multilayered structures utilized by neural networks to extract and transform features from input data. To address this limitation, visualization methods such as Grad-CAM have been increasingly adopted to elucidate the internal decision-making mechanisms of deep neural networks. Consequently, we employed Grad-CAM to analyze the Multiscale Cosine Convolution module, providing insights into the spatial and temporal EEG regions prioritized by each convolutional branch during classification. These visual analyses qualitatively illustrate how individual branches contribute to the final decision and provide representative evidence supporting the interpretability of the MCC-HTSCC model.

Specifically, following the original Grad-CAM formulation [39], we compute Grad-CAM with respect to the last convolutional output of the MCC module (i.e., the feature tensor immediately before the subsequent fusion and classification stages), using the seizure-class logit (pre-softmax) as the target score. In our implementation, the three parallel branches in MCC are concatenated in a width-wise manner, producing a feature map of size 1×256×32. To obtain branch-wise explanations, we split this concatenated feature map sequentially into three equal-width sub-maps of size 1×86×32, corresponding to Branch 1–3, and compute one Grad-CAM heatmap for each sub-map. For a global explanation, we additionally generate a fused heatmap by resizing the three branch-wise maps to the same temporal axis and averaging them.

**Figure 12:**
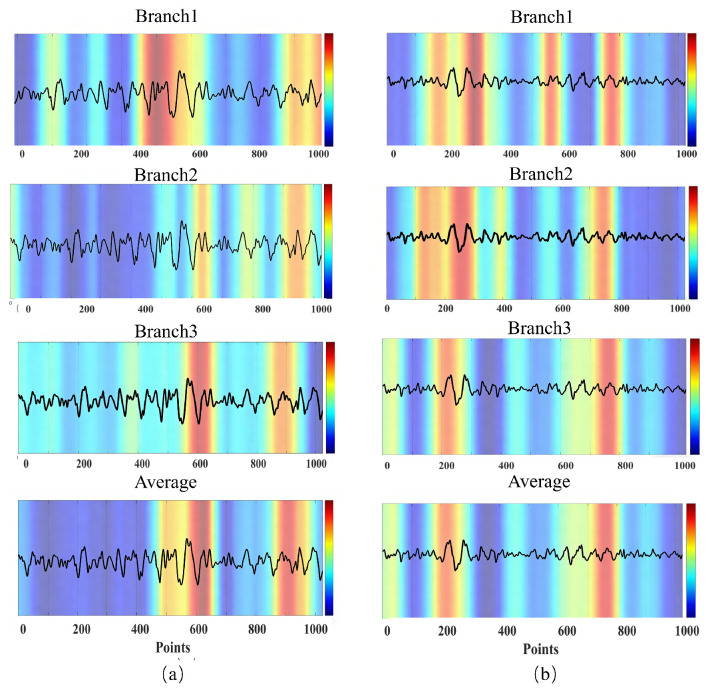
Two representative Grad-CAM visualization examples, illustrating the working principle of the model. (**a**) Grad-CAM visualization of the first representative sample from Patient 12 in the CHB-MIT database; (**b**) Grad-CAM visualization of the second representative sample from Patient 12 in the CHB-MIT database. The color bar indicates the attention level, with red representing higher attention weights and blue representing lower attention weights.

Based on these branch Grad-CAM maps, we further analyzed the critical EEG features emphasized by the three branches of the multiscale cosine convolution module. Through the detailed gradient analysis illustrated in Figure 12, we observed qualitatively that the three branches can exhibit complementary patterns of attention for epileptic seizure detection in representative examples, collectively minimizing the information loss and reducing the likelihood of misclassification. In the representative examples shown here, branch contributions vary across episodes, with one branch often providing the dominant response and the others offering partially overlapping or complementary support when individual branch activation is weaker.

**Figure 13:**
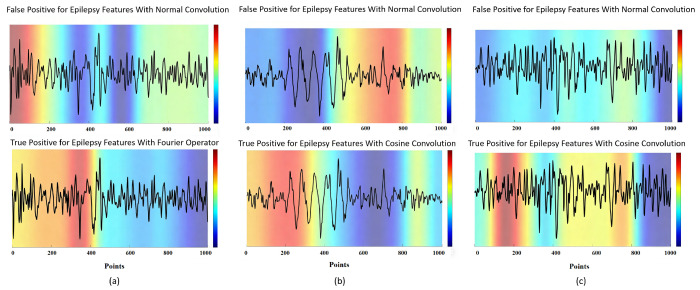
Three representative model visualization examples. (**a**) Representative sample 1 from Patient 12 in the CHB-MIT database; (**b**) representative sample 2 from Patient 12 in the CHB-MIT database; (**c**) representative sample 3 from Patient 12 in the CHB-MIT database. All three samples were misclassified by the model without cosine convolution but were correctly recognized after incorporating cosine convolution, demonstrating the effectiveness of the proposed method. The color bar indicates the attention level, where higher values in red represent higher attention weights and lower values in blue represent lower attention weights.

From these Grad-CAM results, it can be further observed that the proposed model is not designed around any single predefined EEG rhythm band, such as delta, theta, alpha, or beta. Instead, it learns discriminative epileptiform waveform morphologies directly from scalp EEG segments. The Grad-CAM visualizations in Figure 12 and Figure 13 indicate that the most influential regions align with typical seizure-related patterns in the raw traces, including brief high-slope transients resembling spikes or sharp waves, spike and slow wave like composites consisting of a sharp transient, followed by a slower component, and short rhythmic burst-like episodes that appear as clustered oscillations with a higher regularity or amplitude than nearby non-ictal activity. These heatmaps, therefore, provide representative examples of the concrete epileptiform-like waveforms that drive the model’s seizure detection decisions in this study.

Specifically, in the seizure EEG segment illustrated in Figure 12a, Branch 2 mainly focused on the intervals of samples 590–610 and samples 900–1000, both of which contain seizure segments. However, the highlighted regions covered only part of the epileptiform waveforms and failed to reliably identify all seizure-specific EEG patterns when considered alone. If classification relied solely on this branch, such attention deviation could potentially lead to misclassification. In contrast, Branch 1 mainly focused on samples 400–500, an interval containing seizure segments, while Branch 3 mainly focused on samples 580–620, which also contain seizure segments. These two branches successfully captured epileptiform signals with different amplitudes and frequency characteristics, thereby compensating for the incomplete recognition of Branch 2. In addition, both Branch 1 and Branch 3 also showed clear attention to the seizure EEG activity within samples 800–1000, complementing Branch 2 and jointly covering the seizure-related EEG features throughout this period. Consequently, the averaged heatmap further enhances the accurate localization and significance representation of epileptiform EEG features in this critical seizure segment. Similarly, in Figure 12b, although all three branches successfully detected seizure activity, there were still noticeable differences in the temporal intervals emphasized by each branch. In this example, epileptiform waveforms were mainly concentrated in samples 200–300 and samples 750–800. Specifically, Branch 1 mainly focused on samples 250–300, which contain seizure segments, samples 550–600, which do not contain seizure segments, and samples 750–800, which contain seizure segments. Branch 3 mainly focused on samples 180–220 and samples 750–800, both of which also contain seizure segments. Although Branch 1 showed some response to the non-seizure interval of samples 550–600, this deviation was compensated for by the effective attention of Branches 2 and 3 through the averaging fusion of the three branches, allowing the overall model to still correctly identify the true seizure segments. Such complementary attention among branches helps reduce potential false negatives and false positives, since an individual branch operating in isolation may place an emphasis on different signal components.

As illustrated by the two Patient 12 examples in Figure 12, the three branches can attend to partially overlapping yet non-identical temporal regions, and their averaged response provides a more stable localization of seizure-related EEG patterns than any single branch alone. Importantly, the branch-level Grad-CAM behavior observed in these representative examples is not fixed and may vary with seizure morphology and background EEG characteristics. In some patients, Branch 1 may provide the strongest discriminative focus, while Branches 2 and 3 act as complementary cues. In others, Branch 2 or Branch 3 may become more dominant. This qualitative observation is consistent with the design rationale of the proposed multiscale MCC architecture, although a formal cross-patient statistical analysis remains for future work.

Moreover, because the three branches operate in parallel without hierarchical dependencies, they can capture complementary feature patterns at different temporal scales and jointly form a more comprehensive representation of seizure-related EEG activity. As reflected in the Grad-CAM visualizations, this parallel multiscale design appears to provide broader and more stable qualitative coverage of discriminative EEG patterns than any single branch alone. Overall, the multiscale cosine convolution module enhances the network’s ability to characterize seizure-related EEG features across multiple temporal resolutions, while the Grad-CAM results further support the interpretability of the proposed architecture.

Beyond the complementary benefit introduced by the multiscale design, a further advantage lies in the structured cosine parameterization itself, which promotes more selective feature localization. In traditional convolutional neural networks, the convolution kernels learn features through highly unconstrained, weight-based parameterization. Although flexible, this approach often leads to overfitting on non-critical regions when processing structured signals, such as epileptic EEG, which exhibit distinct temporal and spectral characteristics. As demonstrated by the Grad-CAM heatmaps in Figure 13, standard convolution (upper panels) consistently exhibits significant activations within multiple non-epileptiform EEG segments, resulting in frequent false positive identifications. In contrast, cosine convolution (lower panels) imposes structured constraints by parameterizing convolution kernels through cosine functions, requiring only two trainable parameters per kernel. This formulation significantly enhances the model’s inductive bias, enabling greater selectivity in feature extraction and frequency domain responsiveness. The periodic structure inherent to cosine convolution effectively captures characteristic epileptic waveforms, such as spikes and sharp waves, while simultaneously suppressing irrelevant background noise and non-epileptic activities. Consequently, the qualitative visual comparisons suggest that cosine convolution may provide more selective and clinically plausible attention patterns, positioning it as a more reliable and clinically applicable solution for EEG-based seizure detection tasks.

As illustrated in Figure 13a, standard convolution erroneously directs its attention toward regions dominated by high-amplitude noise artifacts, approximately within samples 100–300 and after sample 700, incorrectly interpreting these as epileptic features. In contrast, cosine convolution precisely activates the characteristic spike–wave structure occurring between samples 300–500, effectively eliminating false positive detections by avoiding irrelevant EEG components. Figure 13b further emphasizes this contrast, demonstrating that conventional convolution generates broad, dispersed activations across nearly the entire temporal span. Such diffuse activation reflects poor generalization capability and unclear discriminative boundaries. Conversely, the activation pattern from cosine convolution distinctly focuses on the two prominent EEG waveform peaks appearing within samples 200–500, underscoring its superior spatial compression and feature selectivity. Similarly, for the spike–wave epileptiform activity depicted in Figure 13c, standard convolution displays prominent activations in early and middle EEG segments, approximately within samples 100–400, corresponding primarily to small amplitude high frequency fluctuations lacking genuine epileptic characteristics and thus clearly indicative of misclassification. Cosine convolution, however, accurately localizes its attention on the definitive spike–wave segments between approximately samples 400–700, precisely identifying epileptic events and consequently yielding correct classifications. These visualization analyses strongly substantiate that cosine convolution significantly enhances the model’s ability to accurately and selectively detect epileptic features, thereby substantially reducing false positive rates and improving overall detection reliability in EEG-based seizure detection tasks.

The Grad-CAM visualizations indicate that cosine convolution tends to produce more concentrated activation patterns on seizure-related EEG regions than standard convolution. In the representative examples analyzed, these activations are more closely aligned with characteristic epileptiform waveforms, such as spike and wave or sharp wave patterns. These observations support the interpretability of the proposed model and suggest that cosine parameterization may enhance feature selectivity in EEG-based seizure detection.

### 4.3. Performance Comparisons

The proposed MCC-HTSCC model was rigorously evaluated on the CHB-MIT dataset, which is one of the most widely used benchmarks for patient-specific seizure detection. Table 11 presents a comparison between our method and several representative state-of-the-art (SOTA) models reported in the recent literature. However, we note that these compared results were not obtained under a unified re-implementation. Instead, they were collected from the original papers, and differences in evaluation criteria, the number of used seizures, and patient split strategies, window length/overlap, and post-processing may affect the absolute performance values. Therefore, this table is intended to provide a contextual reference, rather than a strictly controlled head-to-head comparison.

Early deep learning approaches, such as FC-NLSTM [46] and Bi-GRU + Transform [48], reported relatively high segment-based sensitivities of 95.42% and 93.89%, respectively. In contrast, MCC-HTSCC achieves 97.98% segment-based sensitivity and 98.65% event-based sensitivity with a 4 s window. Several methods, such as EMD-CSP + SVM [12] and DTW [49], also achieved competitive performance. Compared with these methods, MCC-HTSCC achieves 98.53% specificity, 98.52% accuracy, and a false detection rate of 0.94/h. Models such as 1D-CNN + RS-DA [47] and ST + CNN [28] reported event-based sensitivities up to 99.31% and 85%, respectively, but their segment-based sensitivities were 88.14% and 79.59%, with false detection rates up to 2.52/h. In comparison, MCC-HTSCC maintains both high segment-based sensitivity and a lower false detection rate of 0.94/h. Recent hybrid architectures, such as TCN-Bi-LSTM [53] and CWT + LightGBM [52], also showed strong performance, with TCN-Bi-LSTM reaching 94.31% segment-based sensitivity and CWT + LightGBM reporting 99.74% segment-based sensitivity. However, CWT + LightGBM lacks event-based evaluation, and our work includes a larger number of seizure events than TCN-Bi-LSTM (184 vs. 115), with even the number of testing events in our study alone exceeding the total number of seizure events reported in TCN-Bi-LSTM (144 vs. 115). MCC-HTSCC also achieves 98.52% accuracy and 98.53% specificity on 979.93 h of EEG data with 184 used seizures. Methods such as OOD [50] and SSDS+DT [51] reported lower performance in Table 11, with OOD achieving 75% segment-based sensitivity, 0.89 specificity, and 0.87 accuracy, while SSDS+DT reported 94.1% sensitivity and 0.975 accuracy without event-based evaluation. Overall, MCC-HTSCC achieves competitive performance across segment-based sensitivity, event-based sensitivity, specificity, accuracy, and false detection rate on the CHB-MIT benchmark.

## 5. Conclusions

In this study, we have introduced MCC-HTSCC, a novel deep learning architecture designed to enhance EEG-based seizure detection by addressing the key limitations of conventional CNNs, including insufficient temporal modeling, high computational complexity, and reduced robustness under limited seizure data. Through comprehensive ablation studies and extensive experiments, we demonstrated that the MCC module captures multiscale temporal dependencies while remaining parameter-efficient and that the HTSCC module extracts hierarchical representations by combining fine-grained local patterns with broader contextual cues, thereby improving detection performance. Evaluations on the CHB-MIT benchmark and our clinically collected SH-SDU dataset show competitive sensitivity, specificity, accuracy, and AUROC.

In terms of practical application, MCC-HTSCC is designed for patient-specific seizure detection in clinical monitoring workflows, such as in hospital long-term monitoring units and bedside surveillance. In these settings, clinicians must review many hours of EEG to localize and quantify seizure events. Our model can automatically screen long recordings and highlight candidate ictal segments for expedited clinician confirmation, thereby reducing manual screening workload and improving review efficiency.

At this stage, our work targets patient-specific seizure detection, while cross-patient seizure detection and seizure prediction remain to be explored. In future work, we will extend the proposed method toward cross-patient seizure detection and further investigate seizure prediction settings. In addition, although MCC-HTSCC has been clinically validated on the publicly available CHB-MIT database and our clinically collected SH-SDU database, it has not yet been deployed on hardware devices for end-to-end real-time testing. Future work will, therefore, expand both clinical databases and embed the algorithm into representative embedded hardware platforms to benchmark practical latency, memory footprint, and power consumption under realistic deployment conditions.

## Figures and Tables

**Table 1:** Patient demographics and seizure statistics.

Patient	Sex	Age	Seizure Type	Seizure Onset Zone	Total Duration (h)	Mean Seizure Duration (s)
1	F	11	SP, CP	Temporal	40.55	63.15
2	M	11	SP, CP, GTC	Frontal	35.27	57.34
3	F	14	SP, CP	Temporal	38.00	57.43
4	M	22	SP, CP, GTC	Temporal, Occipital	156.07	94.50
5	F	7	CP, GTC	Frontal	39.00	111.60
6	F	1.5	CP, GTC	Temporal	66.74	15.30
7	F	14.5	SP, CP, GTC	Temporal	67.05	108.34
8	M	3.5	SP, CP, GTC	Temporal	20.01	183.80
9	F	10	CP, GTC	Frontal	67.87	69.00
10	M	3	SP, CP, GTC	Temporal	50.02	65.50
11	F	12	SP, CP, GTC	Frontal	34.79	268.67
12	F	2	SP, CP, GTC	Frontal	20.69	36.63
13	F	3	SP, CP, GTC	Temporal, Occipital	33.00	44.59
14	F	9	CP, GTC	Temporal	26.00	21.13
15	M	16	SP, CP, GTC	Frontal, Temporal	40.01	99.60
16	F	7	SP, CP, GTC	Temporal	19.00	8.40
17	F	12	SP, CP, GTC	Temporal	21.01	97.67
18	F	18	SP, CP	Temporal, Occipital	35.63	52.84
19	F	19	SP, CP, GTC	Frontal	29.93	78.67
20	F	6	SP, CP, GTC	Temporal	27.60	36.75
21	F	13	SP, CP	Temporal	32.83	49.75
22	F	9	-	Temporal, Occipital	31.00	68.00
23	F	6	-	Frontal	26.56	60.58
24	-	-	-	-	21.30	31.94
Summary	-	-	-	-	979.93	-

Note: GTC = generalized tonic-clonic seizure, CP = complex partial seizures, SP = simple partial seizures.

**Table 2:** Summary of subjects and recording statistics.

Patient	Sex	Age	Total Duration (h)	Mean Seizure Duration (s)	Number of EEG Channels
1	F	28	20.58	40.53	18
2	F	28	23.74	68.8	18
3	M	61	16.04	220.8	18
4	M	34	12	52.38	18
5	M	33	7.24	28.47	18
6	M	72	15.56	105.08	18
7	M	45	6	59.33	18
8	M	71	17.22	795.67	18
9	F	45	26.05	120.06	18
10	F	37	3.8	23.00	18
Summary	–	45.4	148.23	151.412	–

**Table 3:** The result of our proposed system on SH-SDU database with segment-based evaluation criterion.

Patient	Sensitivity	Specificity	Accuracy
1	81.58%	99.22%	99.06%
2	73.05%	83.57%	83.50%
3	88.58%	97.01%	96.67%
4	95.24%	96.85%	96.79%
5	85.90%	99.75%	97.35%
6	87.38%	99.82%	96.74%
7	89.96%	93.05%	92.89%
8	95.08%	99.94%	95.66%
9	100.00%	99.95%	99.79%
10	84.17%	89.77%	87.16%
Average	88.09% ± 9.56%	95.89% ± 7.46%	94.56% ± 8.59%

**Table 4:** The result of our proposed system on SH-SDU database with event-based evaluation criterion.

Patient	Number of Experts-Marked Seizures	Number of Detected Seizures	Sensitivity	FDR (/h)
1	18	12	66.67%	0.28
2	10	7	70.00%	1.98
3	9	9	100.00%	0.19
4	9	8	88.89%	1.67
5	19	19	100.00%	0.14
6	8	6	87.50%	0.08
7	28	28	100.00%	1.42
8	37	37	100.00%	0.06
9	2	2	100.00%	0.00
10	3	3	100.00%	0.41
Average	143	131	91.31% ± 12.36%	0.62 ± 0.71

**Table 5:** The result of our proposed system on the CHB-MIT database with a segment-based evaluation criterion.

Patient	Sensitivity	Specificity	Accuracy
1	100.00%	99.71%	99.71%
2	100.00%	99.72%	99.72%
3	100.00%	99.07%	99.07%
4	100.00%	97.56%	97.56%
5	100.00%	99.95%	99.95%
6	100.00%	96.99%	96.99%
7	100.00%	99.92%	99.92%
8	94.27%	95.14%	95.09%
9	100.00%	100.00%	100.00%
10	100.00%	99.97%	99.97%
11	100.00%	99.92%	99.92%
12	98.32%	97.31%	97.24%
13	81.11%	98.19%	98.14%
14	100.00%	96.70%	96.70%
15	98.97%	98.86%	98.85%
16	100.00%	91.34%	91.29%
17	100.00%	99.92%	99.92%
18	87.50%	99.70%	99.67%
19	100.00%	99.92%	99.92%
20	98.63%	97.10%	97.04%
21	100.00%	99.85%	99.85%
22	100.00%	99.99%	99.99%
23	100.00%	99.70%	99.70%
24	92.65%	98.28%	98.24%
Average	97.98% ± 4.60%	98.53% ± 2.01%	98.52% ± 2.03%

**Table 6:** The result of our proposed system on the CHB-MIT database with an event-based evaluation criterion.

Patient	Number of Seizures Experts Marked	Number of Seizures Detected	Sensitivity	FDR (/h)
1	6	6	100.00%	0.02
2	2	2	100.00%	0.23
3	6	6	100.00%	0.32
4	3	3	100.00%	0.22
5	4	4	100.00%	0.03
6	6	6	100.00%	3.30
7	2	2	100.00%	0.1
8	4	4	100.00%	0.65
9	3	3	100.00%	0.01
10	5	5	100.00%	0.02
11	2	2	100.00%	0
12	23	23	100.00%	1.7
13	8	7	87.50%	0.76
14	7	7	100.00%	2.54
15	19	19	100.00%	0.20
16	2	2	100.00%	10.65
17	2	2	100.00%	0.05
18	5	4	80.00%	0.06
19	2	2	100.00%	0
20	7	7	100.00%	0.62
21	3	3	100.00%	0.03
22	2	2	100.00%	0
23	6	6	100.00%	0.11
24	15	15	100.00%	1.08
Average	144	142	98.61% ± 4.62%	0.95 ± 2.18

**Table 7:** Average performance by seizure type on the CHB-MIT database.

Seizure Type	Sensitivity	Specificity	Event-Based Sensitivity	Accuracy	FDR (/h)
SP, CP	96.88%	99.58%	95.00%	99.58%	0.11
SP, CP, GTC	97.79%	98.07%	99.04%	98.04%	1.16
CP, GTC	100.00%	98.41%	100.00%	98.41%	1.47

Note: Each entry reports the average performance across the patients belonging to the corresponding seizure-type group.

**Table 8:** The effect of the number of branches.

Model	Parameters	AUC
1-Branch(CosCNN) + 2HTSCC	35 k	0.883
2-Branch(CosCNN) + 2HTSCC	57.5 k	0.911
3-Branch(CosCNN) + 2HTSCC	80 k	0.932
4-Branch(CosCNN) + 2HTSCC	99.9 k	0.919
5-Branch(CosCNN) + 2HTSCC	125 k	0.898

Note: Increasing the number of parallel cosine convolution branches improves AUC from 0.883 to a peak of 0.932.

**Table 9:** The effect of the number of HTSCC modules.

Model	Parameters	AUC
3-Branch(CosCNN) + 1HTSCC	76.9 k	0.897
3-Branch(CosCNN) + 2HTSCC	80 k	0.936
3-Branch(CosCNN) + 3HTSCC	92.5 k	0.931
3-Branch(CosCNN) + 4HTSCC	99.9 k	0.926

Note: Using two HTSCC modules achieves the best AUC of 0.936.

**Table 10:** The effect of the CosCNN module.

Model	Configuration	AUC	Parameters
3-Branch(CosCNN) + 2HTSCC	1, 5, 9	0.936	80 k
	5, 9, 13	0.928	80 k
3-Branch(CNN) + 2HTSCC	1, 5, 9	0.932	97.7 k
	5, 9, 13	0.927	97.7 k

Note: Cosine convolution reduces parameters from 97.7 k (CNN) to 80 k (CosCNN) while maintaining comparable AUC.

**Table 11:** Comparison of different methods for epileptic seizure detection (statistics are taken from the original papers. Protocols may differ across methods).

No.	Author (Year)	Method	Window Length	Length of EEG Data Used	Number of Used Seizure	Sensitivity (Segment-Based/ Event-Based)	Specificity	Accuracy	FDR (/h)
1	Li et al. (2020) [46]	FC-NLSTM	4 s	846.23	198	95.42%/95.29%	95.29%	-	0.66
2	Wang et al. (2021) [47]	1D-CNN + RS-DA	2 s	916 h	198	88.14%/99.31%	99.62%	99.54%	0.2
3	Li et al. (2021) [12]	EMD-CSP + SVM	2 s	976.9 h	185	97.34%/98.47%	97.50%	-	0.63
4	Zhang et al. (2022) [48]	Bi-GRU + Transform	4 s	870.44 h	198	93.89%/95.49%	98.49%	98.49%	0.63
5	Sopic et al. (2023) [49]	DTW	1s	996 h	198	96%/90.4%	-	-	0
6	Wong et al. (2023) [50]	OOD	1 s	916 h	198	75%/-	89.00%	87.00%	0.94
7	Wang et al. (2024) [51]	SSDS + DT	1 s	-	-	94.1%/-	87.60%	97.50%	-
8	Saranya et al. (2024) [52]	CWT + LightGBM	4 s	-	-	99.74%/-	98.26%	98.53%	-
9	Dong et al. (2024) [53]	TCN-Bi-LSTM	4 s	820.26	115	94.31%/96.48%	97.13%	97.09%	0.38
10	Liu et al. (2025b) [28]	ST + CNN	4 s	979.93 h	184	79.59%/85%	92.23%	93.45%	2.52
11	This work	MCC-HTSCC	4 s	979.93 h	184	97.98%/98.65%	98.53%	98.52%	0.94

## Data Availability

The data are available from the corresponding author upon reasonable request, subject to ethical and privacy restrictions.

## Abbreviations

The following abbreviations are used in this manuscript:

EEG	Electroencephalogram
MCC	Multiscale Cosine Convolution
HTSCC	Heterogeneous Two-Stream Cosine Convolution
MCC-HTSCC	Multiscale Cosine Convolutional Heterogeneous Two-Stream Cosine Convolution Network
CosCNN	Cosine Convolutional Neural Network
CNN	Convolutional Neural Network
FCN	Fully Convolutional Network
FC-NLSTM	Fully Convolutional Nested Long Short-Term Memory
LSTM	Long Short-Term Memory
Bi-LSTM	Bidirectional Long Short-Term Memory
GRU	Gated Recurrent Unit
Bi-GRU	Bidirectional Gated Recurrent Unit
TCN	Temporal Convolutional Network
STFT	Short-Time Fourier Transform
FFT	Fast Fourier Transform
CWT	Continuous Wavelet Transform
DWT	Discrete Wavelet Transform
Db4	Daubechies-4 wavelet
ST	Stockwell Transform (S-transform)
ASTFT	Adaptive Short-Time Fourier Transform
SST	Synchrosqueezing Transform
MAF	Moving Average Filter
CSP	Common Spatial Pattern
SCSP	Sparse Common Spatial Pattern
PSD	Power Spectral Density
SVM	Support Vector Machine
EMD	Empirical Mode Decomposition
DTW	Dynamic Time Warping
PCA	Principal Component Analysis
LightGBM	Light Gradient Boosting Machine
OOD	Out-of-Distribution
MSA-DCNN	Multi-Scale Atrous-based Deep Convolutional Neural Network
Grad-CAM	Gradient-weighted Class Activation Mapping
t-SNE	t-distributed Stochastic Neighbor Embedding
ROC	Receiver Operating Characteristic
AUC	Area Under the Curve
AUROC	Area Under the Receiver Operating Characteristic Curve
FDR	False Detection Rate
CHB-MIT	Children’s Hospital Boston–MIT EEG Database
SH-SDU	Second Hospital of Shandong University EEG Database
